# Prescribing clozapine and rifampicin: clinical impact of their interaction

**DOI:** 10.1192/pb.bp.115.051250

**Published:** 2016-06

**Authors:** Caroline Parker

**Affiliations:** 1 Central and North West London NHS Foundation Trust

## Abstract

The predictable pharmacokinetic drug interaction between clozapine and rifampicin is listed in most standard reference texts but little detail is given or emphasis on its clinical significance. The interaction is based on theoretical knowledge of both drugs; to date just two case reports have been published. This article describes a third case demonstrating the significance of this interaction. This was potentially devastating for the patient who required an extended psychiatric admission. The enzyme induction was so potent that the dose of clozapine had to be increased approximately sixfold. Careful management of this significant interaction is essential for effective patient care.

Clozapine was the first second-generation antipsychotic. It has superior efficacy to other antipsychotics,^1^ however, owing to its propensity to cause blood dyscrasias its use is restricted; in the UK it is licensed for use in treatment-resistant schizophrenia and psychosis during Parkinson's disease.^2^ Clozapine is not a narrow-therapeutic-window drug, but the measurement of serum levels has been established as beneficial in optimising the dose, assessing adherence and minimising dose-related adverse effects.^3–5^ The dose corresponding to therapeutic serum levels varies considerably and is affected by gender, cigarette smoking and age as well as concurrent interacting medicines.^4^ People can vary dramatically in the rate at which they metabolise clozapine (up to 50-fold slower/faster).^6^

Like most antipsychotics clozapine is extensively metabolised via the hepatic cytochrome P450 system. The main metabolite via the CYP1A2 isoenzyme, norclozapine, is pharmacologically active, with other isoenzymes involved (including 2C19, 2D6, 2C9 and 3A4) producing more minor metabolites. Extensive reviews of clozapine's pharmacokinetics and interactions are available.^7–9^

Rifampicin, introduced to the UK in 1967, is licensed for the treatment of infections including tuberculosis.^10^ It is a potent inducer of cytochrome P450 including the isoenzymes CYP1A2 and 3A4. It is widely recognised as interacting with numerous medicines metabolised via these routes,^10,11^ and its pharmacokinetics and interactions have also been extensively reviewed.^12–15^

The interaction between clozapine and rifampicin is listed in most standard texts,^6,11,16–18^ although not all.^5,10^ Even when mentioned, there is little detail on its significance. The *British National Formulary* (BNF)^11^ states ‘rifampicin possibly reduces plasma concentration of clozapine’, but does not class it as significant or give any guidance regarding the management of this interaction. This article describes a case demonstrating this interaction.

## Clinical case report

A 30-year-old female with paranoid schizophrenia had been taking clozapine for the past 8 years with good efficacy and minimal side-effects. She remained mentally stable in the community, adhered to blood tests, and there were no haematological concerns. She was a smoker, but had not misused illicit drugs or alcohol since she was in her early 20s. Over the past 2 or 3 years she had developed skin problems, including quite severe acne on her face. She held some paranoid delusions about clozapine so at her request during the previous year her maintenance dose had been gradually reduced over a number of months, down to 100 mg daily, augmented with amisulpride (for residual symptoms).

Approximately 3 months before her psychiatric admission she had seen a dermatologist. They had avoided prescribing isotretinoin (Roaccutane) (presumably due to the warnings that it can, albeit rarely, cause psychiatric symptoms including depression, mood alterations, psychosis and suicidal ideation)^19^ and started rifampicin 300 mg twice daily. Subsequently she had an acute and severe psychotic relapse. Both her clozapine and norclozapine were less than 0.05 mg/L, i.e. not meaningfully detectable. Approximately a week after admission rifampicin was stopped and replaced with clindamycin which does not interact with clozapine.^2,20^ However, her presentation continued to deteriorate. Over the following weeks the clozapine was increased to 350 mg/day, but levels were subtherapeutic (clozapine 0.28 mg/L, norclozapine 0.15 mg/L) and she only began to show some initial signs of improvement after a month. The clozapine dose was further increased to 400 mg/day, yet levels remained subtherapeutic (clozapine 0.20 mg/L, norclozapine 0.10 mg/L) and consequently the dose was further increased to 425 mg/day. Over 2–3 months her presentation gradually improved, the dose continued to be titrated up (levels were just therapeutic on 550 mg/day: clozapine 0.40 mg/L, norclozapine 0.14 mg/L) to 650 mg/day, without side-effects.

## Discussion

Although this particular interaction is discussed in a number of reviews,^8,9,12,15,21–24^ it is predominantly based on one case report from 1998^25^ and a few refer to a second case published nearly a decade later.^26^ In the first case,^25^ a 33-year-old male with schizophrenia who had a very good response to clozapine developed tuberculosis and was treated with rifampicin 600 mg daily, isoniazid and pyrazinamide. Within 2–3 weeks his clozapine serum levels decreased approximately sixfold and were subtherapeutic, and at 3.5 weeks psychotic symptoms returned. Prescribers increased his clozapine from 400 mg to 600 mg/day, but this did not lead to any improvement. Rifampicin was then substituted with ciprofloxacin, and the clozapine reduced back to 400 mg/day. Three days later clozapine serum levels returned to therapeutic values and the patient's psychotic symptoms improved. The second case^26^ was a man with schizophrenia who was stable and well on clozapine 300 mg/day; rifampicin 600 mg/day was started for tuberculosis. Two weeks later his psychotic symptoms worsened and his previous clozapine dose-related adverse effects ceased. His clozapine was increased to 550 mg/day with only mild improvement. Six months later the rifampicin was stopped and his psychotic symptoms improved significantly and the previous dose-related adverse effects re-emerged. No clozapine levels were reported. In both cases the patient's previous clozapine dose-related adverse effects ceased when rifampicin was administered and their psychotic symptoms returned. Increasing the clozapine dose was not sufficient to manage the interaction.^18^

In another case^27^ where this combination was prescribed concurrently for 9 months the pharmacokinetic interaction was not mentioned, and although the patient did relapse, the main focus was the fact that they developed late-onset neutropenia following the introduction of rifampicin. Prior to that he had been mentally and haematologically stable on clozapine for 9 years.

Given these limited reports of this pharmacokinetic interaction, the description in standard texts is based on the theoretical knowledge of both drugs. This article describes a third case demonstrating the significance of this interaction, where clozapine was rendered ineffective by the potent enzyme induction of rifampicin. The serious psychotic relapse that ensued led to the patient's first psychiatric admission in about 8 years.

Generally, enzyme induction interactions are slow to develop and slow to resolve. Induction may take several days or 2–3 weeks to fully develop;^13,17^ the extent of enzyme induction depends on both the drug and its dose.^14^ The induction may persist for a similar period after stopping the inducer, although this varies considerably between drugs.^14,18^ Rifampicin's onset effect on clozapine is thought to be 2–3 weeks,^18,25^ and induction is near maximal at 300 mg/day.^13^

The case described here was in keeping with these facts. The impact of the interaction on the patient's presentation was clinically significant at about 3 months. Although rifampicin's effect on the enzymes was likely to have lasted for at least 1–2 weeks after the drug was discontinued,^12,18^ the effect on the patient's clinical presentation took much longer to resolve. It could have been anticipated that the patient's clozapine maintenance dose (100 mg daily) would not lead to therapeutic serum levels but unfortunately none were recorded in that or the preceding year. Following the introduction and cessation of rifampicin, the patient's dose of clozapine was increased approximately sixfold, suggesting that the enzyme induction was very potent. From an interaction perspective, an alternative antipsychotic that is not induced by rifampicin^18^ could have been considered, rather than persisting with clozapine in the face of this interaction. However, this option was not pursued as the patient's schizophrenia was treatment resistant; several alternative antipsychotics had previously been tried but the patient failed to respond to them. Therefore, the decision was to optimise the clozapine dose carefully using therapeutic levels as a guide, aware that on withdrawal of an enzyme inducer plasma concentrations of the induced drug may increase and toxicity may occur.^11^

In the Summary of Product Characteristics (SPC) for rifampicin the only antipsychotics identified as interacting with it are haloperidol and aripiprazole,^10^ although lurasidone and clozapine are also mentioned in the BNF.^11^

The incidence of tuberculosis in England had increased steadily from the late 1980s to 2005, and has remained at relatively high levels ever since. England has one of the highest tuberculosis rates in Western Europe. The incidence is more than four times higher than in the USA. If current trends continue, England will have more tuberculosis cases than the whole of the USA within 2 years.^28^ Therefore it is anticipated that there will be more patients in whom both clozapine and rifampicin are indicated, and this will need to be carefully managed.

## Conclusions and implications for clinical practice

This is the third known case demonstrating the impact on a patient of a pharmacokinetic drug interaction between clozapine and rifampicin. Despite the predictability of this reaction from the theoretical knowledge of these established medicines, there is little in the literature to give prescribers advice in managing it. Although efforts have been made to systematically assess the causality of adverse drug reactions, these do not take into account direct pharmacokinetic interactions. When rated using the Adverse Drug Reaction Probability Scale,^29^ a reaction such as that observed in this case study (loss of antipsychotic control) would be considered to be a ‘probable’ adverse drug reaction.

There is a need for controlled studies to establish the importance and extent of such reactions.^12^ Clinicians are reminded and encouraged to report all drug interactions that lead to severe adverse effects to the Medicines and Healthcare products Regulatory Agency via the Yellow Card Scheme.^30^ Prescribers are also encouraged to heed the significance of this particular interaction, and to work across team interfaces so that psychiatrists liaise with pharmacists and pulmonologists to carefully review prescriptions to optimise patient care.
